# Correlation of the total superoxide dismutase activity between joint fluid and synovium in end-stage knee osteoarthritis

**DOI:** 10.1038/s41598-024-62614-x

**Published:** 2024-05-27

**Authors:** Masato Koike, Hidetoshi Nojiri, Hiroaki Kanazawa, Mamiko Sawa, Kei Miyagawa, Hiroto Yamaguchi, Yoshiyuki Iwase, Hisashi Kurosawa, Kazuo Kaneko, Muneaki Ishijima

**Affiliations:** 1 Department of Orthopaedic Surgery, Juntendo Tokyo Koto Geriatric Medical Center, Tokyo, Japan; 2 Department of Orthopaedics, Juntendo University Faculty of Medicine, Tokyo, Japan

**Keywords:** Cartilage, End-stage knee osteoarthritis, Joint fluid, Superoxide dismutase activity, Synovial membrane, Cell biology, Biomarkers

## Abstract

Recently, we found significantly reduced total superoxide dismutase (SOD) activity in the cartilage of patients with end-stage knee osteoarthritis (OA). In this study, we aimed to evaluate the SOD activity in serum, joint fluid, cartilage, and synovial membrane samples collected from 52 patients with end-stage knee OA who underwent total knee arthroplasty. The relationship between the total SOD activity in each tissue was evaluated using Spearman’s rank correlation coefficient. The joint fluid total SOD activity was used as the objective variable, and its association with the serum, cartilage, and synovial total SOD activities was evaluated using multiple linear regression analysis. Univariate analysis revealed that joint fluid total SOD activity was positively correlated with synovial total SOD activity. Multiple linear regression analysis using joint fluid total SOD activity as the objective variable showed a positive association with synovial total SOD activity (*β* = 0.493, adjusted *R*^2^ = 0.172, *P* < 0.01). In patients with end-stage knee OA, the state of the synovial total SOD activity is better reflected by the total SOD activity in the joint fluid than that in the cartilage. Joint fluid total SOD activity may serve as a biomarker for the treatment and prevention of synovitis.

## Introduction

Superoxide dismutase (SOD) is an enzyme that eliminates the harmful molecule superoxide. SOD1, SOD2, and SOD3 are localized in the cytoplasm and mitochondria and outside the cell, respectively. Between 2006 and 2010, an association between osteoarthritis (OA) and SOD was reported by three separate groups. The first group reported that SOD2 expression was significantly lower in the articular cartilage in knee OA than in the femoral head of patients with femoral neck fractures^1^. The second group used proteomic analysis to demonstrate that the SOD2 protein level was significantly reduced in the articular cartilage in knee OA compared with that in the femoral head of patients with femoral neck fractures^2^. In the third group, DNA chip analysis revealed that *SOD2* expression was significantly reduced in the articular cartilage of patients with knee OA^3^. However, whether the decrease in SOD levels is a result of OA remains unclear, and the underlying mechanisms are poorly understood. Our previous study revealed that *Sod2*-depleted chondrocytes accelerated cartilage degeneration due to excessive mechanical stress and aging in chondrocyte-specific *Sod2*-deficient mice^4^ and that oxidative stress was a cause of cartilage degeneration. Furthermore, we showed a strong relationship between end-stage OA and decreased SOD activity^5^.

OA is a chronic progressive joint disease that primarily involves cartilage degeneration. Over 25.3 million patients aged ≥ 40 years have OA in Japan. Hence, markers related to the pathophysiology of OA are needed to enable an early diagnosis and prediction of its course, although the course of OA is slow, and the nature and stages of the disease are heterogeneous. Clinical trials often have insufficient sample sizes and durations to demonstrate treatment efficacy. Although biomarkers that are detectable in body fluids, such as urine, plasma, serum, and joint fluids, have attracted attention^6^, no useful biomarkers have yet been identified. SOD has been revealed as a potential biomarker for other diseases^7^. Therefore, we focused on SOD as a potential biomarker of OA.

Some groups have reported a relationship between SOD activity in the serum and joint fluid of patients with OA^8^ and proposed a relationship between joint fluid SOD and viscosity^9^. However, no reports have clarified the correlation between systemic and local SOD activity by simultaneously measuring SOD activity in the cartilage and synovium. Predicting the total SOD activity in cartilage and synovial tissues based on the total SOD activity values in the serum and joint fluid will enable the noninvasive determination of the suitability of OA treatment and prevention.

The present study aimed to simultaneously measure systemic total SOD activity (serum) and intra-articular total SOD activity (joint fluid, articular cartilage, and synovium) in patients with end-stage knee OA and to clarify the relationship between systemic and joint total SOD activity.

## Results

Baseline data for 52 patients who underwent total knee arthroplasty (TKA) were as follows: 5 males and 47 females with an average age of 75.3 years, average body mass index (BMI) of 25.3 kg/m^2^, and OA with a Kellgren–Lawrence (KL) grade of 4 (Table 1, Supplementary Table S1).

**Table 1:** Baseline characteristic of the patients with end-stage knee OA.

Characteristics	Total (*n* = 52)
Sex (male/female)	5/47
Age (years)	75.3 ± 7.0
BMI (kg/m^2^)	25.3 ± 4.4
KL grade	Grade 4

Values are indicated as mean ± standard deviation.

BMI, body mass index; OA, osteoarthritis; KL, Kellgren–Lawrence.

**Figure 1:**
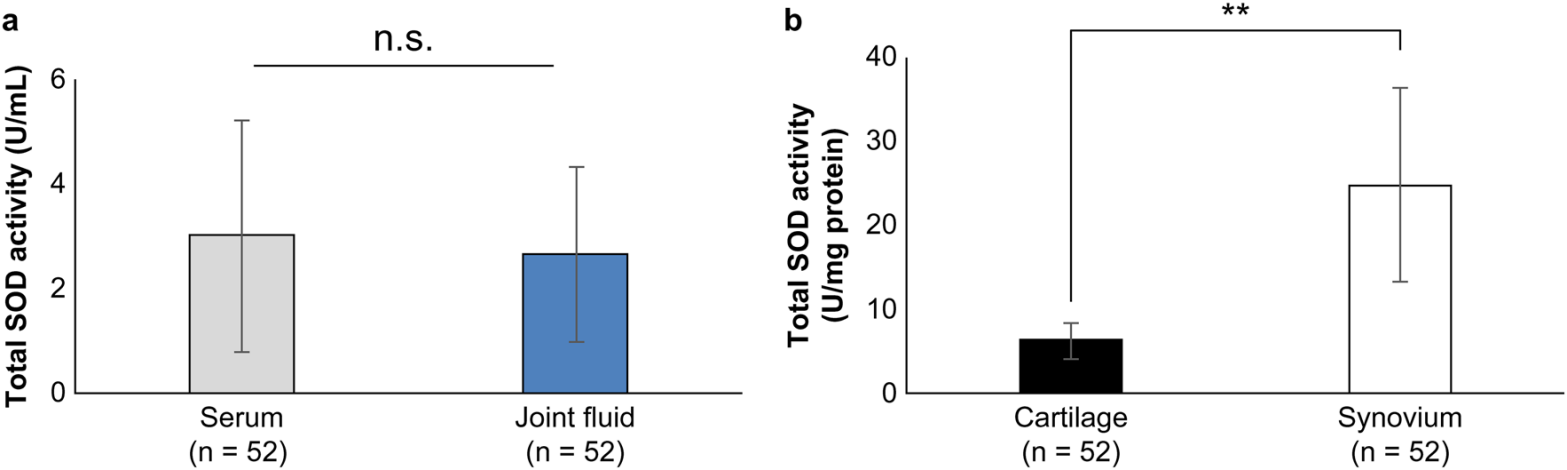
Total superoxide dismutase (SOD) activity in the serum, joint fluid, cartilage, and synovium of patients with end-stage knee osteoarthritis (OA). (**a**) Comparison of serum total SOD activity and joint fluid total SOD activity in patients with end-stage knee OA (*n* = 52, n.s.: not significant, paired t-test). (**b**) Comparison of cartilage total SOD activity and synovial total SOD activity in patients with end-stage knee OA (*n* = 52, **P < 0.01, paired t-test).

We compared the whole-body total SOD activity with the intra-articular total SOD activity. No significant differences were observed between the total SOD activity in the serum and joint fluid (Fig. 1a). Furthermore, the total SOD activity was significantly higher in the joint fluid than in the cartilage (Fig. 1b, Supplementary Table S2).

**Figure 2:**
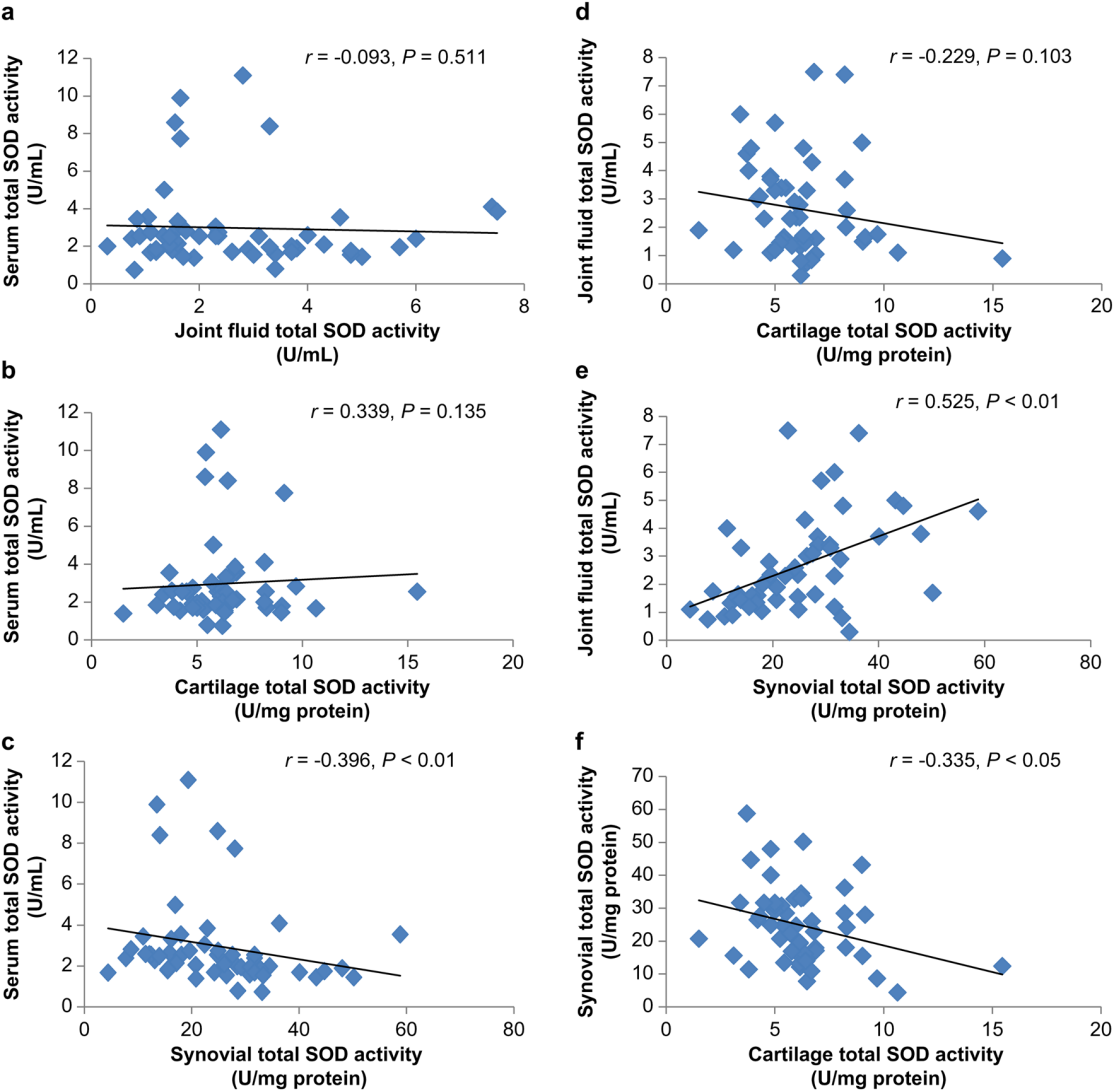
Correlation of total superoxide dismutase (SOD) activities in end-stage knee osteoarthritis (OA). (**a**) Correlation between serum total SOD activity and joint fluid total SOD activity (*n* = 52, Spearman’s correlation). (**b**) Comparison of serum total SOD activity and cartilage total SOD activity (*n* = 52, Spearman’s correlation). (**c**) Comparison of serum total SOD activity and synovial total SOD activity (*n* = 52, Spearman’s correlation). (**d**) Comparison of joint fluid total SOD activity and cartilage total SOD activity (*n* = 52, Spearman’s correlation). (**e**) Comparison of joint fluid total SOD activity and synovial total SOD activity (*n* = 52, Spearman’s correlation). (**f**) Comparison of synovial total SOD activity and cartilage total SOD activity (*n* = 52, Spearman’s correlation).

Univariate analysis was performed to assess the correlation between the serum, joint fluid, cartilage, and synovium. Individual data on the total SOD activity in the serum, joint fluid, cartilage, and synovium in patients with end-stage knee OA are presented in Supplementary Table S2. The normality of the data was confirmed, and the correlation of each tissue was evaluated using Spearman’s rank correlation coefficient. No correlation was observed between the following: serum total SOD activity and joint fluid total SOD activity (Fig. 2a), serum total SOD activity and cartilage total SOD activity (Fig. 2b), and the total SOD activities of the joint fluid and cartilage (Fig. 2d). A negative correlation was observed between the serum total SOD activity and synovial total SOD activity (*r* =  − 0.396, *P* < 0.01) (Fig. 2c), as well as between the cartilage total SOD activity and synovial total SOD activity (*r* =  − 0.335, *P* < 0.05) (Fig. 2f). A positive correlation was observed between the joint fluid total SOD activity and synovial total SOD activity (*r* = 0.525, *P* < 0.01) (Fig. 2e).

Multiple regression analysis was performed using the joint fluid total SOD activity as the objective variable and the serum, cartilage, and synovial total SOD activities as explanatory variables. Multiple linear regression analysis using the joint fluid total SOD activity as the objective variable showed a positive association with the synovial total SOD activity (*β* = 0.493, adjusted *R*^2^ = 0.172, *P* < 0.01) (Tables 2, 3). The state of the synovial total SOD activity was better reflected by the joint fluid total SOD activity than the cartilage total SOD activity in patients with end-stage knee OA.

**Table 2:** Multivariate analysis. Multivariate linear analysis with joint fluid total SOD activity as the objective variable.

Multiple linear regression analysis	Unstandardized coefficient		Standardized coefficient	*t*-value	*P*-value
	B	Standard error	β		
(Constant)	0.641	3.019		0.212	0.833
Serum total SOD activity (U/mL)	0.035	0.101	0.046	0.345	0.732
Cartilage total SOD activity (U/mg protein)	0.017	0.109	0.022	0.153	0.879
Synovial total SOD activity (U/mg protein)	0.072	0.020	0.493	3.587	0.001
Age (years)	0.015	0.032	0.063	0.481	0.633
BMI (kg/m^2^)	− 0.045	0.052	− 0.116	− 0.866	0.391

Objective variable: joint fluid total SOD activity (U/mL).

Explanatory variables: serum total SOD activity, cartilage total SOD activity, and synovial total SOD activity.

BMI, body mass index; SOD, superoxide dismutase.

**Table 3:** Multivariate analysis. Model summary of multivariate linear analysis with joint fluid total SOD activity as the objective variable.

Model summary				
	*R*	*R*^2^	Adjusted *R*^2^	Standard error
Model	0.503	0.253	0.172	1.53309

We also conducted an analysis for females only, but the trend of the correlation remained the same (data not shown). Because there were no gender differences, analyses were performed for males and females.

## Discussion

The results of this study showed that the total SOD activity in the joint fluid was positively correlated with the total SOD activity in the synovium in patients with end-stage knee OA.

SOD undergoes various post-translational modifications, including nitration, glycation, glutathionylation, and phosphorylation. Changes in gene expression and protein levels do not necessarily directly reflect the enzyme activity, as proteins can undergo post-translational modifications^10^. Furthermore, in a rat model, SOD2 activity reportedly decreased owing to an increase in lysine acetylation, a post-translational modification; in contrast, the amount of the SOD2 protein in cartilage increased with age^11^. Based on these findings, we focused on assessing the total SOD activity rather than the gene expression and protein abundance.

In this study, as a preliminary experiment to determine whether to evaluate the protein levels or activity, we measured the protein levels of SOD1, which is localized in the cytoplasm, and of SOD2, which is localized in the mitochondria of cartilage and synovial tissues. We then evaluated the relationship between SOD1 levels and total SOD activity in the cartilage and synovium, respectively. We also evaluated the relationship between SOD2 levels and SOD2 activity in the cartilage and synovium, respectively (Supplementary Fig. S1). The cartilage total SOD activity had a strong positive correlation with the cartilage SOD1 protein level (Supplementary Fig. S1). The synovial total SOD activity had a moderate positive correlation with the synovial SOD1 protein level (Supplementary Fig. S1). Furthermore, the synovial SOD2 activity had a moderate positive correlation with the synovial SOD2 protein level. The cartilage SOD2 activity also had a moderate positive correlation with the cartilage SOD2 protein level (Supplementary Fig. S1). In the cartilage, the mean SOD1 level was 2.22 µg/mg protein, whereas the mean SOD2 level was 0.20 µg/mg protein. In the synovium, the mean SOD1 level was 3.86 µg/mg protein, whereas the mean SOD2 level was 1.83 µg/mg protein (Supplementary Fig. S2, Supplementary Tables S3, S4). Both tissues had a comparatively higher SOD1 protein content, indicating that the cytoplasm accounted for a larger proportion than the mitochondria. In the cartilage, the mean total SOD activity level was 5.00 U/mg protein, whereas the mean SOD2 activity level was 0.80 U/mg protein. In the synovium, the mean total SOD activity level was 30.90 U/mg protein, whereas the mean SOD2 activity level was 18.70 U/mg protein (Supplementary Fig. S2, Supplementary Tables S3, S4). These results suggest that SOD undergoes post-translational modifications and that changes in gene expression and protein levels do not necessarily directly reflect its activity^10^. Based on these results, we evaluated the activity rather than the protein level. Regan et al. have suggested that the total SOD activity in OA is dominated by SOD1 activity and may not reflect the activity in specific extracellular compartments where SOD3 has unique binding affinities; furthermore, separation is difficult to perform accurately and varies greatly, depending on the method used^12^.

Mice lacking SOD3 display more severe arthritis than that of wild-type mice. SOD3 has been reported to be crucial in rheumatoid arthritis^13^. Although decreased SOD3 has been demonstrated in the cartilage of patients with OA, its role in the pathology has not been well studied. Thus, based on the above findings, we chose to evaluate the total SOD activity rather than to specifically evaluate SOD3 activity.

Our previous study reported significantly lower total SOD activity in the cartilage and synovium of patients with end-stage knee OA than that of controls; moreover, the total SOD activity was lower in both the cartilage and synovium but tended to be higher in the synovium than in the cartilage^5^. In this study, the total SOD and SOD2 activities were significantly higher in the synovium than in the cartilage (Supplementary Fig. S1). As the total SOD and SOD2 activities in the cartilage were depleted owing to cartilage wear, the synovial membrane, which retains total SOD activity, may function as a biomarker. The joint fluid comprises hyaluronic acid tissue fluid from B cells of the synovial surface and ultrafiltered plasma fluid^14^. Therefore, we focused on the total SOD activity of the joint fluid related to the synovium. Regarding the SOD activity in OA, Sumii et al. reported that the joint fluid SOD activity was higher than the serum SOD activity in patients with primary and secondary knee OA^8^. In addition, Ostalowska et al. reported that the joint fluid SOD activity was significantly higher in patients with knee OA than in patients with joint effusion who did not have OA^9^. In both reports, the joint fluid SOD activity was higher than the serum SOD activity. In this study, no significant difference (Fig. 1a) or correlation (Fig. 2a) was observed between the serum and joint fluid total SOD activities. Kalogera et al. reported several biomarkers that showed significantly higher values in the serum than in the joint fluid and concluded that their effects on the whole body must be considered^15^. Based on past reports^8,9^ and the present results, we believed that evaluations using joint fluid rather than serum would be more appropriate because the SOD activity may be associated with systemic metabolism. Melon GliSODin® (i.e., melon extract with a high SOD content), an oral supplement containing SOD extracted from melon and wheat gliadin, activates the antioxidant enzyme SOD and has beneficial effects in various diseases^16^. Our previous 6-month double-blind study on patients with pain or discomfort in their knees or lower back revealed that the serum total SOD activity did not change before and after GliSODin and placebo administration in both the oral GliSODin and placebo groups, respectively^17^. The pain and discomfort tended to improve after drug administration. Furthermore, joint injection of vitamin C derivatives into chondrocyte-specific *Sod2*-deficient mice suppressed cartilage degeneration^4^. Therefore, monitoring changes in the total SOD activity in the joints rather than in the serum is important. Monitoring intra-articular total SOD activity changes rather than serum total SOD activity changes may serve as a biomarker for the treatment and prevention of cartilage degeneration and synovitis. Therefore, in this study, we conducted the analysis using the joint fluid total SOD activity as the objective variable.

In this study, we performed a multiple linear regression analysis using the joint fluid total SOD as the objective variable, excluding the effects of age and BMI, which are confounding factors. The total SOD activity in the joint fluid was positively correlated with the synovial total SOD activity. However, no correlation was observed in the total SOD activity between the joint fluid and cartilage. Given the strong association between the joint fluid and synovium, assessing the total SOD activity of the joint fluid may predict the degree of the total SOD activity in the synovium. Recently, Gui et al. reported that the intra-articular injection of highly efficient antioxidant nanoparticles based on SOD-loaded porous polymersome nanoparticles (SOD-NPs) into mice undergoing OA surgery inhibited the development of OA^18^. OA onset was reduced, and OA progression was prevented. SOD-NP accumulated in the synovium of mouse joints but not in the articular cartilage. Therefore, targeting the synovium holds great promise for OA treatment, suggesting that the future target for OA treatment and prevention may shift to the synovium rather than the cartilage. Clinically, we hope to control OA by evaluating the total SOD activity in the joint fluid collected via arthrocentesis at the outpatient clinic. Until now, most treatments for OA have targeted cartilage. The results of this study suggest that total SOD activity is more greater in the synovium than that in the cartilage, and that targeting the synovium for treatment or prevention may suppress OA progression.

This study has two main limitations. First, the cartilage and synovial tissues were collected from outside the patellofemoral joint, which is a non-load-bearing area. Because changes in OA noticeably occur in loaded areas, the difference in total SOD activity between the loaded and non-loaded areas should be evaluated. However, in load-bearing areas with a KL grade of 4, most of the cartilage is worn out or absent, and evaluating the load-bearing area in patients with end-stage knee OA is difficult. The second drawback in this study is that we evaluated the total SOD activity in the final stage of OA. To explore biomarkers for the onset and prevention of progression of OA, serum, joint fluid, and cartilage samples from patients with early knee OA are required. However, collecting cartilage and synovial tissues from patients with early knee OA is highly invasive, and evaluating patients with early knee OA is unethical and impractical. The method of measuring the total SOD activity differs among research groups that have reported in the past, and the different measurement methods may be affected by systemic metabolism. Thus, future studies will be required to evaluate the total SOD activity between the loaded and non-loaded area for each grade of OA, and evaluating this method is necessary.

In conclusion, the joint fluid total SOD activity in patients with end-stage knee OA is positively correlated with the synovial total SOD activity, and the joint fluid total SOD activity better reflects the synovial condition than the cartilage total SOD activity. New findings may be obtained by investigating arthritis with joint effusion and comparing the different grades of OA.

## Methods

### Ethical approval

The study protocol was approved by the Faculty of Medicine Research Ethics Committee of Juntendo University, Japan (approval number: E23-0368). All experiments were conducted in accordance with the principles of the Declaration of Helsinki. Informed consent was obtained from all patients before the study.

### Collection and preservation of tissue samples

Serum, joint fluid, cartilage, and synovium samples were obtained from the knee joints of patients who underwent TKA for end-stage knee OA. The degree of OA was evaluated using the KL classification^19^. The exclusion criteria for all patients were a history of rheumatoid arthritis, local joint infection, or gout.

Blood was collected on the morning of the surgery. Blood was centrifuged at 1500 × *g* for 5 min after blood collection and immediately stored at − 80 °C, as previously described^17^.

Joint fluid was collected from the lateral part of the patellofemoral joint using an 18 G needle before making a skin incision during surgery. Joint fluid was frozen and stored at − 80 °C after the surgery.

Samples of the cartilage and synovium were taken from outside the patellofemoral joint using biopsy forceps, as previously described^5^. Cartilage and synovium samples were stored at − 80 °C immediately after collection.

### Sample preparation before measuring total SOD activity and SOD2 activity and SOD1 and SOD2 protein expression

Before SOD measurement, the serum and joint fluid were thawed at room temperature, and the supernatant was collected without centrifugation. The difference in the total SOD activity of the joint fluid with and without centrifugation is shown in Supplementary Fig. S3.

Before SOD measurements, the cartilage and synovium were thawed at room temperature, and 300 μL of a physiological phosphate-buffered solution and two stainless steel balls were placed in a bead beater-type homogenizer (Beads Crusher μT-01; TAITEC Co., Ltd., Saitama, Japan). Specimens were then homogenized twice at 4600 rpm for 30 s. The homogenate was collected and centrifuged for 10 min at 4 °C and 10,000 × *g*. The supernatant was collected.

### Measuring total SOD activity in serum, joint fluid, cartilage, and synovium

The total SOD activity in the samples was analyzed using the Superoxide Dismutase Assay Kit (#706002, Cayman Chemicals, Ann Arbor, MI, USA) and the tetrazolium method, which measures superoxide radicals produced by xanthine oxidase using a tetrazolium salt, according to the manufacturer’s instructions, as previously described^5^. The absorbance was measured at 450 nm using an Infinite M 200 microplate reader (Tecan Group, Ltd., Männedorf, Switzerland).

### Measuring SOD2 activity in serum, joint fluid, cartilage, and synovium

The addition of potassium cyanide (KCN) inhibits SOD (SOD1) and extracellular SOD (SOD3) in activity assays^20^. SOD2 activity in cartilage and synovial tissue homogenates was detected using the Superoxide Dismutase Assay Kit (#706002, Cayman Chemicals, Ann Arbor, MI, USA) and the tetrazolium method, according to the manufacturer’s protocol. To inactivate SOD1 and SOD3, KCN (Nacalai Tesque, Inc., Kyoto, Japan) was added to the reaction mixture and adjusted to a final concentration of 10 mM. Thereafter, the mixture was incubated at room temperature for 30 min, and the absorbance at 450 nm was measured using an Infinite M 200 microplate reader (Tecan Group, Ltd., Männedorf, Switzerland).

### Total SOD activity and SOD2 expression in serum and joint fluid

The serum and joint fluid, which are liquids, have a constant concentration, which was expressed in U per mL, without protein correction. Thus, the total activities of SOD and SOD2 in the serum and joint fluid were expressed in U per mL.

### Total SOD activity and SOD2 expression in cartilage and synovium

SOD undergoes various post-translational modifications, such as nitration, glycation, glutathionylation, and phosphorylation^10^. Therefore, the gene expression or protein levels may not correlate with the activity. Therefore, the activity was evaluated by adjusting for the amount of protein in the solid tissues. As previously reported, the cartilage and synovium were evaluated in U per mg of protein^5^. Protein levels were determined using the Wako Protein Assay Rapid Kit (Wako Pure Chemical Industries, Ltd., Osaka, Japan), according to the manufacturer’s instructions. Using a microplate, 10 μL of the sample and 250 μL of the coloring reagent were thoroughly mixed and heated at 37 °C for 30 min. Absorbance at 600 nm was monitored using a Tecan Microplate Reader Infinite M 200 (Tecan Group, Ltd., Männedorf, Switzerland), and the protein concentration was calculated from the calibration curve. The total SOD and SOD2 activities in the cartilage and synovium were expressed as U per mg protein.

### Measuring SOD1 protein in cartilage and synovium

To quantify SOD1 in the cartilage and synovium, the RayBio® Human SOD1 ELISA kit was used. This in vitro enzyme-linked immunosorbent assay used antibodies specific to human SOD1 coated onto 96-well plates. All reagents, samples, and standards were prepared according to the manufacturer’s instructions. The samples were diluted 150 times using 1 × assay diluent. A 500 mg/mL standard solution was prepared by dissolving the SOD1 protein in 1 × assay diluent. According to the manufacturer’s instructions, a dilution series of 12,000, 4,800, 1,920, 768, 307.2, 122.9, and 49.15 pg/mL was prepared, and the 1 × assay dilution was used as a zero standard (0 pg/mL). The detection antibody solution was diluted 80-fold using 1 × assay diluent buffer. The horseradish peroxidase (HRP)-streptavidin solution was prepared by diluting the HRP-streptavidin concentrate 700 times with 1 × assay diluent buffer. Next, 100 μL of each standard and sample were added to the wells, covered, and incubated for 2.5 h at room temperature with gentle shaking. The solution was aspirated, and the wells were washed four times by filling each well with 300 μL of 1 × washing buffer. The solution was aspirated from the wells, and the plate was inverted to completely remove the solution. Next, 100 μL of biotinylated antibody prepared at a 1 × dilution was added to each well and incubated for 1 h at room temperature with gentle shaking. The solution was aspirated, and the wells were washed four times by filling each well with 300 μL of 1 × washing buffer. The solution was aspirated from the wells, and the plate was inverted to completely remove the solution. Next, 100 μL of streptavidin solution was added to each well and incubated for 45 min at room temperature with gentle shaking. The solution was aspirated, and the wells were washed four times by filling each well with 300 μL of 1 × washing buffer. The solution was aspirated from the wells, and the plate was inverted to completely remove the solution. Finally, 100 μL of 3,3’,5,5’-tetramethylbenzidine (TMB) One-Step Substrate Reagent was added to each well and incubated for 30 min at room temperature in the dark with gentle shaking, followed by the addition of 50 μL of 0.2 M sulfuric acid as a stop solution. The absorbance was measured at 450 nm using an Infinite M 200 microplate reader (Tecan Group, Ltd., Männedorf, Switzerland).

### Measuring SOD2 protein in cartilage and synovium

SOD2 in the cartilage and synovium was quantified using the SOD2 (Human) ELISA Kit (Abnova, Atlanta, GA, USA) according to the manufacturer’s instructions.

First, the solutions for SOD2 quantification were prepared. A 50 ng/mL standard stock solution was prepared by adding 1 mL of standard/sample dilution buffer to lyophilized recombinant human SOD2 protein. The solution was incubated at room temperature for 5 min and mixed thoroughly by gentle vortexing. One milliliter of a 5,000 pg/mL top standard was prepared, and two-fold serial dilutions of the 5,000 pg/mL top standards were performed to generate a standard curve within the range of this assay (78.125–5000 pg/mL). The standard/sample dilution buffer was used as the zero standard (0 pg/mL). Standard solutions of 5,000, 2,500, 1,250, 625, 312.5, 156.25, 78.125, and 0 pg/mL were established. The secondary antibody solution was prepared by diluting 100 × with secondary antibody/avidin-linked HRP (Av-HRP) dilution buffer. The washing buffer was prepared by diluting the wash buffer concentrate solution 20 × with deionized water.

Next, 300 µL of incubation buffer was added to each well of the 96-well plates coated with a mouse monoclonal antibody specific to human SOD2 and incubated for 5 min at room temperature. Thereafter, the solution was aspirated from each well, and the wells were washed twice. For the standard curve, 100 μL of the standard and 100 μL of standard/sample dilution buffer was added to the wells. The samples were diluted 20-fold in standard/sample dilution buffer, and 100 μL of the samples was added to each well. The sample (100 μL) diluted 20 times in standard/sample dilution buffer was added to each well. The plate was covered and incubated for 2 h at room temperature. The solution was completely aspirated from the wells, and the wells were washed three times with washing buffer. Next, 100 μL of a secondary antibody solution was pipetted into each well, and the plate was covered and incubated for 1 h at room temperature. The solution was aspirated thoroughly from the wells and was washed three times with a washing buffer. Then, 100 μL of Av-HRP solution was added to each well. The plate was covered and incubated for 30 min at room temperature. The solution was aspirated, and the wells were washed three times. Finally, TMB solution was added to each well and incubated at room temperature. When the wells turned blue, 100 μL of 1 N sulfuric acid solution was added to each well as a stop solution. When the color of the solution in the well changed from blue to yellow, the absorbance was monitored at 450 nm using an Infinite M 200 microplate reader (Tecan Group, Ltd., Männedorf, Switzerland).

The total SOD activity, SOD2 activity, and SOD1 and SOD2 levels were measured at the Japan Institute for the Control of Aging and NIKKEN SEIL Co. Ltd. (Shizuoka, Japan).

### Statistical analysis

All statistical analyses were performed using SPSS Statistics version 28 (IBM Corp., Armonk, NY, USA). Data are expressed as mean ± standard deviation. Statistical significance was defined as *P* < 0.05. A paired t-test was used to compare the total SOD activity between the serum and joint fluid and between the cartilage and synovium. No previous reports were usable as a reference, and estimating the effect size was difficult. Assuming the "moderate" effect size proposed by Cohen in 1988^21^, we used the G*Power software to calculate the sample size using an effect size of 0.25, power of 0.8, significance level of 0.05, and 3 explanatory variables. The sample size was calculated as 52, assuming an outlier of approximately 10%.

#### Univariate analysis

To evaluate the relationship between each tissue and the joint fluid, the correlations between the serum total SOD activity, cartilage total SOD activity, synovial total SOD activity, and joint fluid total SOD activity were evaluated using univariate analysis. Data showing correlations between two variables were checked to determine whether they followed a normal distribution. Pearson’s correlation coefficient was used when data were normally distributed. When data did not follow a normal distribution, Spearman’s rank correlation analysis was used.

#### Multivariate analysis

Multiple linear regression analysis (forced entry method) was used, with the joint fluid total SOD activity as the objective variable (dependent variable) and the total SOD activities of the serum, cartilage, and synovium as explanatory variables (independent variables).

### Supplementary Information

Supplementary Figure 1.

Supplementary Figure 2.

Supplementary Figure 3.

Supplementary Tables.

Supplementary Legends.

## Data Availability

All relevant data are within the paper and its Supplementary files.
