# Supplementary figures and images for: Correlation of the total superoxide dismutase activity between joint fluid and synovium in end-stage knee osteoarthritis
Source: Sci Rep. 2024 May 27;14:12093. doi: 10.1038/s41598-024-62614-x (PMC11130189; doi:10.1038/s41598-024-62614-x)

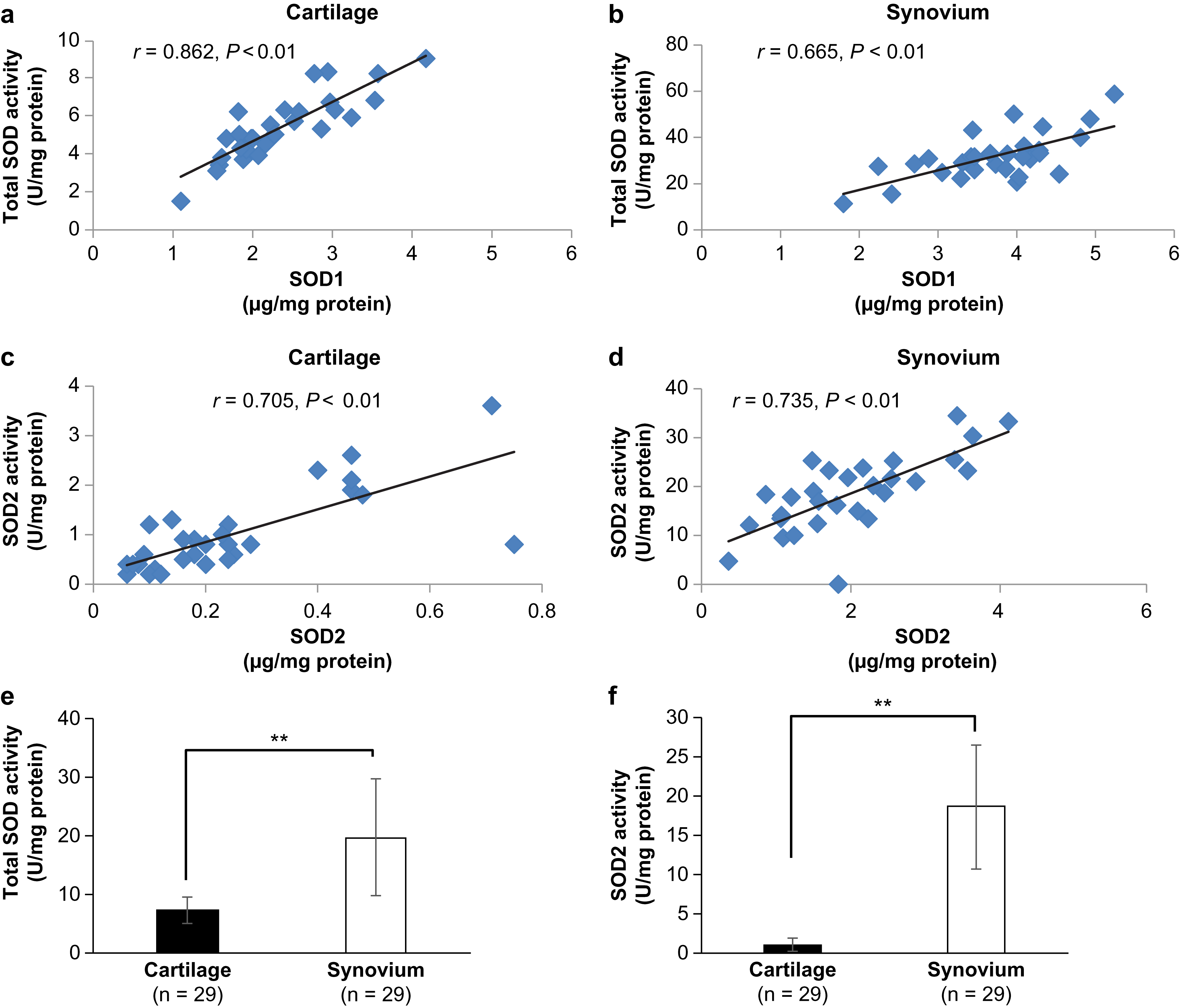

Supplement: Supplementary file 1 — Supplementary Figure 1. [file 41598_2024_62614_MOESM1_ESM.tif]

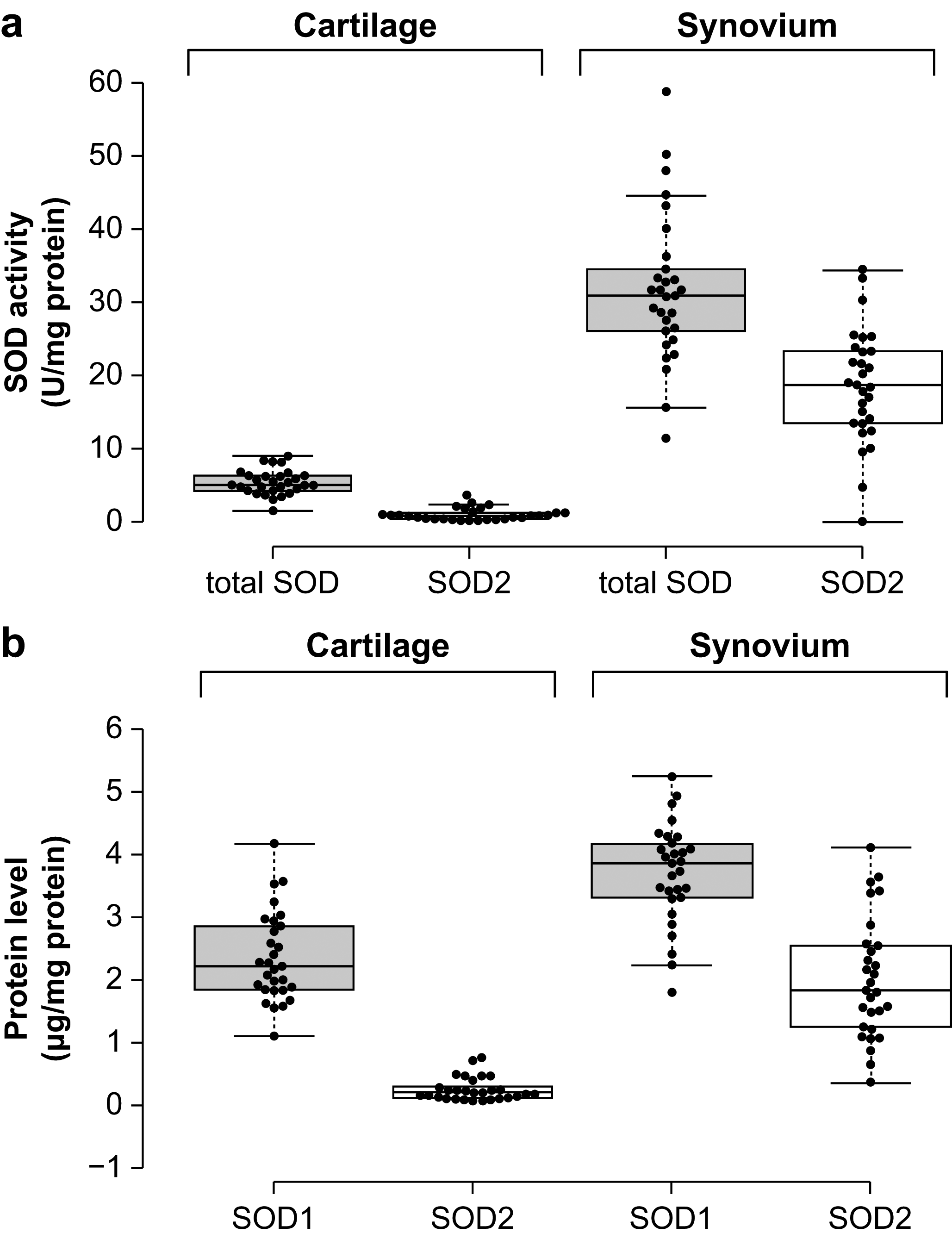

Supplement: Supplementary file 2 — Supplementary Figure 2. [file 41598_2024_62614_MOESM2_ESM.tif]

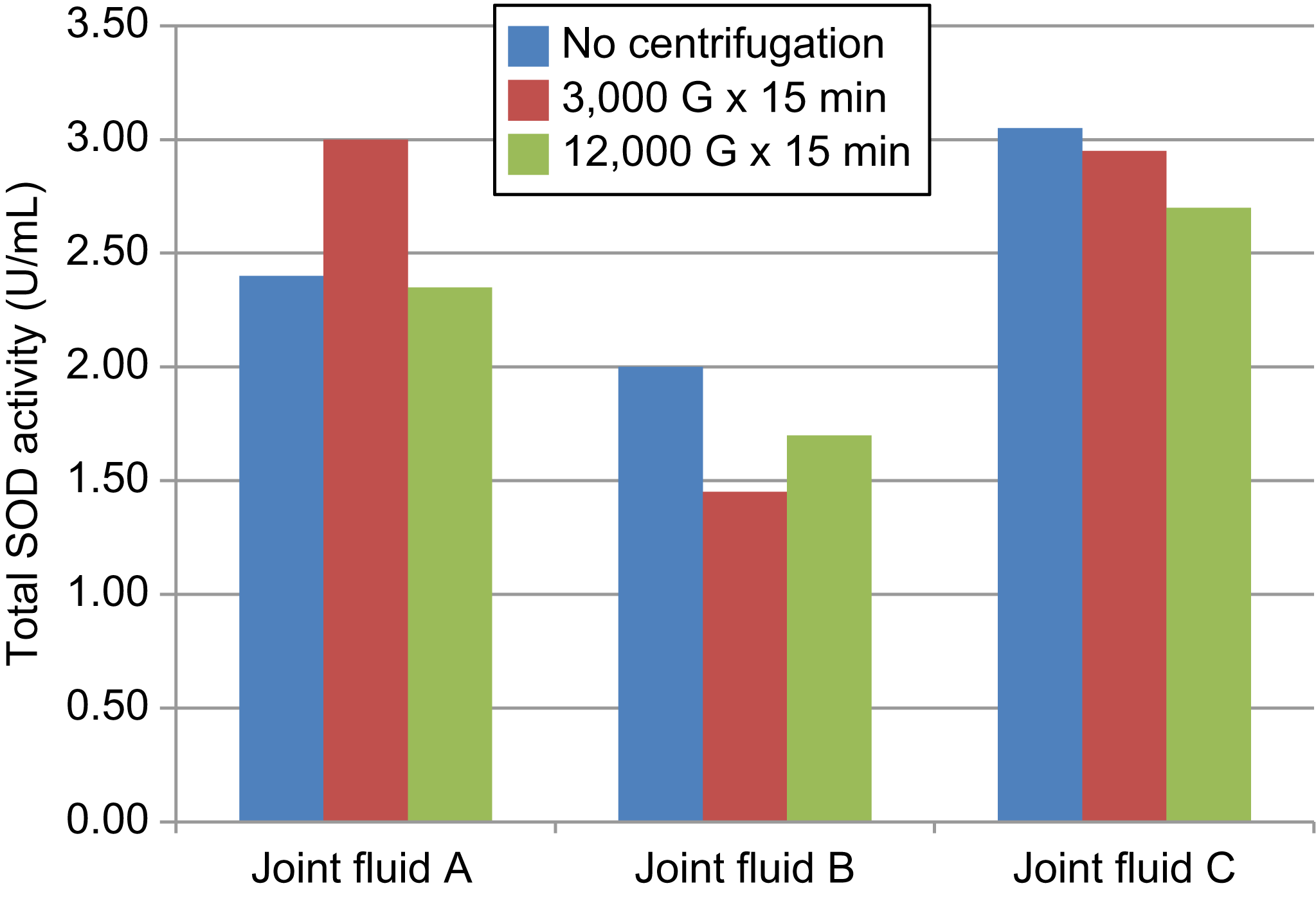

Supplement: Supplementary file 3 — Supplementary Figure 3. [file 41598_2024_62614_MOESM3_ESM.tif]
