# Supplementary material for: Correlation of the total superoxide dismutase activity between joint fluid and synovium in end-stage knee osteoarthritis
Source: Sci Rep. 2024 May 27;14:12093. doi: 10.1038/s41598-024-62614-x (PMC11130189; doi:10.1038/s41598-024-62614-x)
Supplement: Supplementary file 4 — Supplementary Tables. [file 41598_2024_62614_MOESM4_ESM.docx]

**Supplementary Tables**

**Correlation of the total superoxide dismutase activity between joint fluid and synovium in end-stage knee osteoarthritis**

Masato Koike^1,2^, Hidetoshi Nojiri^1,2*^, Hiroaki Kanazawa^1,2^, Mamiko Sawa^1,2^, Kei Miyagawa^1,2^, Hiroto Yamaguchi^1,2^, Yoshiyuki Iwase^1,2^, Hisashi Kurosawa^1,2^, Kazuo Kaneko^2^, Muneaki Ishijima^2^

*** Correspondence** and requests for materials should be addressed to Hidetoshi Nojiri, MD, PhD (Email: [hnojiri@juntendo.ac.jp](mailto:hnojiri@juntendo.ac.jp))

**Supplementary Table 1.** Individual data on the baseline characteristics of patients with end-stage knee osteoarthritis (OA)

| **No.** | **Sex** | **Age**  **(years)** | **Height**  **(cm)** | **Weight**  **(kg)** | **Body mass index**  **(kg/m^2^)** | **Kellgren–Lawrence grade** |
| --- | --- | --- | --- | --- | --- | --- |
| 1 | Male | 88 | 154.2 | 42.4 | 17.8 | 4 |
| 2 | Female | 76 | 146 | 50.2 | 23.6 | 4 |
| 3 | Female | 79 | 160 | 54 | 21.1 | 4 |
| 4 | Female | 76 | 147.5 | 59.45 | 27.3 | 4 |
| 5 | Female | 66 | 158 | 60 | 24 | 4 |
| 6 | Female | 58 | 154 | 53 | 22.3 | 4 |
| 7 | Female | 80 | 153 | 69 | 29.5 | 4 |
| 8 | Female | 73 | 148.5 | 54 | 24.5 | 4 |
| 9 | Female | 87 | 158 | 67.6 | 27.1 | 4 |
| 10 | Male | 76 | 165 | 68 | 25 | 4 |
| 11 | Female | 75 | 159.3 | 57.8 | 22.8 | 4 |
| 12 | Female | 79 | 150 | 64.5 | 28.7 | 4 |
| 13 | Female | 72 | 157 | 42 | 17 | 4 |
| 14 | Female | 67 | 157 | 55 | 22.3 | 4 |
| 15 | Female | 73 | 155.5 | 55.4 | 22.9 | 4 |
| 16 | Female | 79 | 155 | 66 | 27.5 | 4 |
| 17 | Female | 74 | 150 | 70 | 31.1 | 4 |
| 18 | Female | 71 | 155 | 73.6 | 30.6 | 4 |
| 19 | Female | 72 | 151.2 | 70.6 | 30.9 | 4 |
| 20 | Female | 71 | 147.5 | 73.5 | 33.8 | 4 |
| 21 | Female | 86 | 148 | 45 | 20.5 | 4 |
| 22 | Male | 73 | 163 | 72 | 27.1 | 4 |
| 23 | Female | 70 | 155 | 59 | 24.6 | 4 |
| 24 | Female | 82 | 145.3 | 79.1 | 37.5 | 4 |
| 25 | Female | 69 | 154.7 | 54.85 | 22.9 | 4 |
| 26 | Male | 83 | 160.5 | 61.2 | 23.8 | 4 |
| 27 | Female | 61 | 155.8 | 54.1 | 22.3 | 4 |
| 28 | Female | 81 | 150 | 45.9 | 20.4 | 4 |
| 29 | Female | 74 | 151 | 67 | 29.4 | 4 |
| 30 | Female | 63 | 157 | 62 | 25.2 | 4 |
| 31 | Female | 69 | 157 | 55 | 22.3 | 4 |
| 32 | Male | 87 | 153 | 68.75 | 29.4 | 4 |
| 33 | Male | 78 | 161.5 | 64.9 | 24.9 | 4 |
| 34 | Female | 76 | 148.2 | 60.2 | 27.4 | 4 |
| 35 | Female | 81 | 149 | 55 | 24.8 | 4 |
| 36 | Male | 68 | 175 | 80 | 26.1 | 4 |
| 37 | Female | 78 | 148 | 51.8 | 23.6 | 4 |
| 38 | Female | 84 | 145 | 53.5 | 25.4 | 4 |
| 39 | Male | 77 | 161 | 74.95 | 28.9 | 4 |
| 40 | Female | 71 | 158 | 35.2 | 14.1 | 4 |
| 41 | Female | 71 | 158 | 76.1 | 30.5 | 4 |
| 42 | Female | 76 | 159 | 59.8 | 23.7 | 4 |
| 43 | Female | 72 | 150 | 66.6 | 29.6 | 4 |
| 44 | Female | 82 | 150 | 47.3 | 21 | 4 |
| 45 | Female | 72 | 148 | 62.8 | 28.7 | 4 |
| 46 | Female | 79 | 145 | 45.8 | 21.8 | 4 |
| 47 | Male | 69 | 158 | 64 | 25.6 | 4 |
| 48 | Female | 68 | 155 | 76 | 31.6 | 4 |
| 49 | Female | 81 | 162.5 | 61 | 23.1 | 4 |
| 50 | Female | 86 | 151 | 55 | 24.1 | 4 |
| 51 | Female | 71 | 155 | 60 | 25 | 4 |
| 52 | Female | 88 | 146 | 40.2 | 18.9 | 4 |

**Supplementary Table 2.** Individual data of serum total SOD activity, joint fluid total SOD activity, cartilage total SOD activity, and synovial total SOD activity in patients with end-stage knee osteoarthritis (OA).

| **No.** | **Serum** | **Joint fluid** | **Cartilage** | **Synovial** |
| --- | --- | --- | --- | --- |
|  | **total SOD activity** | **total SOD activity** | **total SOD activity** | **total SOD activity** |
|  | **(U/mL)** | **(U/mL)** | **(U/mg protein)** | **(U/mg protein)** |
| 1 | 1.8 | 1.5 | 9 | 15.5 |
| 2 | 11.1 | 2.8 | 6.1 | 19.3 |
| 3 | 2.7 | 1.5 | 6.1 | 15.9 |
| 4 | 2.6 | 2 | 8.3 | 18.1 |
| 5 | 2.6 | 2.4 | 6 | 24.8 |
| 6 | 7.8 | 1.7 | 9.1 | 28 |
| 7 | 3.6 | 1.1 | 6.9 | 18 |
| 8 | 1.7 | 1.1 | 10.6 | 4.4 |
| 9 | 2.6 | 1.3 | 6.2 | 12.1 |
| 10 | 2.2 | 1.6 | 6.9 | 17.1 |
| 11 | 9.9 | 1.7 | 5.4 | 13.5 |
| 12 | 8.4 | 3.3 | 6.5 | 14.1 |
| 13 | 8.6 | 1.6 | 5.4 | 24.8 |
| 14 | 2.4 | 0.8 | 6.5 | 7.8 |
| 15 | 2.8 | 2.4 | 6.1 | 19.6 |
| 16 | 2.8 | 1.8 | 9.7 | 8.7 |
| 17 | 3.5 | 0.9 | 6.7 | 11 |
| 18 | 2.3 | 1.6 | 6.1 | 13.1 |
| 19 | 3.3 | 1.6 | 6.4 | 16.2 |
| 20 | 2.6 | 0.9 | 15.5 | 12.4 |
| 21 | 5 | 1.4 | 5.8 | 16.9 |
| 22 | 2.1 | 1.5 | 5.2 | 20.8 |
| 23 | 2.5 | 1.5 | 6.4 | 14 |
| 24 | 1.7 | 2.6 | 8.3 | 24.2 |
| 25 | 4.1 | 7.4 | 8.2 | 36.3 |
| 26 | 1.9 | 3.8 | 4.8 | 48 |
| 27 | 1.5 | 5 | 9 | 43.2 |
| 28 | 2 | 3.7 | 8.2 | 28.5 |
| 29 | 2.8 | 1.1 | 4.8 | 24.9 |
| 30 | 0.8 | 3.4 | 5.5 | 28.6 |
| 31 | 2 | 0.3 | 6.2 | 34.5 |
| 32 | 3.6 | 4.6 | 3.7 | 58.8 |
| 33 | 1.6 | 3.4 | 5.3 | 30.8 |
| 34 | 2.4 | 6 | 3.4 | 31.7 |
| 35 | 1.9 | 2.9 | 5.9 | 32.8 |
| 36 | 2 | 5.7 | 5 | 29.2 |
| 37 | 1.6 | 3 | 4.2 | 26.5 |
| 38 | 2 | 3.3 | 5 | 30.9 |
| 39 | 3.1 | 2.3 | 5.7 | 22.4 |
| 40 | 1.4 | 1.9 | 1.5 | 20.8 |
| 41 | 1.7 | 1.2 | 5 | 31.7 |
| 42 | 1.7 | 3.7 | 4.8 | 40.1 |
| 43 | 2.1 | 4.3 | 6.7 | 26.1 |
| 44 | 2.6 | 4 | 3.8 | 11.4 |
| 45 | 1.9 | 1.2 | 3.1 | 15.6 |
| 46 | 3.9 | 7.5 | 6.8 | 22.9 |
| 47 | 1.6 | 4.8 | 6.3 | 33.3 |
| 48 | 0.8 | 0.8 | 6.2 | 33.1 |
| 49 | 1.8 | 4.8 | 3.9 | 44.7 |
| 50 | 2.6 | 2.3 | 4.5 | 31.7 |
| 51 | 1.5 | 1.7 | 6.3 | 50.2 |
| 52 | 2.6 | 3.1 | 4.3 | 27.6 |

**Supplementary Table 3.**  Statistical results of box plots in supplementary Fig.2a and b

**a. Results of the five statistics of the box plot in Fig 2a**

|  | **Cartilage**  **total SOD activity**  **(U/mg protein)** | **Cartilage**  **SOD2 activity**  **(U/mg protein)** | **Synovial**  **total SOD activity**  **(U/mg protein)** | **Synovial**  **SOD2 activity**  **(U/mg protein)** |
| --- | --- | --- | --- | --- |
| Upper whisker | 9.00 | 2.30 | 44.70 | 34.50 |
| 3rd quartile | 6.30 | 1.20 | 34.50 | 23.30 |
| Median | 5.00 | 0.80 | 30.90 | 18.70 |
| 1st quartile | 4.30 | 0.40 | 26.10 | 13.50 |
| Lower whisker | 1.50 | 0.20 | 15.60 | 0.00 |
| Nr. of data points | 29.00 | 29.00 | 29.00 | 29.00 |

**b. Results of the five statistics of the box plot in Fig 2b**

|  | **Cartilage SOD1**  **(µg/mg protein)** | **Cartilage SOD2**  **(µg/mg protein)** | **Synovial SOD1**  **(µg/mg protein)** | **Synovial SOD2**  **(µg/mg protein)** |
| --- | --- | --- | --- | --- |
| Upper whisker | 4.17 | 0.48 | 5.24 | 4.11 |
| 3rd quartile | 2.86 | 0.28 | 4.17 | 2.54 |
| Median | 2.22 | 0.20 | 3.86 | 1.83 |
| 1st quartile | 1.85 | 0.11 | 3.31 | 1.24 |
| Lower whisker | 1.10 | 0.06 | 2.24 | 0.36 |
| Nr. of data points | 29.00 | 29.00 | 29.00 | 29.00 |

**Supplementary Table 4.** Individual data of cartilage total superoxide dismutase (SOD) activity, synovial total SOD activity, cartilage SOD2 activity, synovial SOD2 activity, cartilage SOD1 protein, cartilage SOD2 protein, synovium SOD1 protein, and synovium SOD2 protein in patients with end-stage knee osteoarthritis (OA)

|  | **Cartilage** | **Synovium** | **Cartilage** | **Synovium** | **Cartilage** | | **Synovium** | |
| --- | --- | --- | --- | --- | --- | --- | --- | --- |
| **No.** | **total SOD activity** | | **SOD2 activity** | | **SOD1** | **SOD2** | **SOD1** | **SOD2** |
|  | **(U/mg protein)** | | **(U/mg protein)** | | **(µg/mg protein)** | | | |
| 1 | 9.00 | 15.50 | 0.90 | 12.10 | 2.94 | 0.18 | 4.54 | 0.64 |
| 2 | 6.10 | 19.30 | 1.90 | 20.20 | 3.57 | 0.46 | 4.09 | 2.30 |
| 3 | 6.10 | 15.90 | 0.60 | 18.70 | 1.67 | 0.25 | 4.93 | 2.45 |
| 4 | 8.30 | 18.10 | 3.60 | 33.30 | 4.17 | 0.71 | 3.44 | 4.11 |
| 5 | 6.00 | 24.80 | 0.80 | 13.50 | 2.77 | 0.75 | 3.73 | 1.06 |
| 6 | 9.10 | 28.00 | 0.30 | 9.50 | 1.98 | 0.11 | 3.05 | 1.09 |
| 7 | 6.90 | 18.00 | 0.40 | 19.00 | 2.22 | 0.20 | 2.70 | 1.50 |
| 8 | 10.60 | 4.40 | 0.20 | 23.80 | 1.82 | 0.10 | 4.28 | 2.16 |
| 9 | 6.20 | 12.10 | 0.60 | 21.60 | 1.88 | 0.18 | 5.24 | 2.54 |
| 10 | 6.90 | 17.10 | 0.80 | 25.30 | 2.86 | 0.24 | 4.17 | 1.48 |
| 11 | 5.40 | 13.50 | 1.20 | 10.00 | 1.58 | 0.24 | 4.08 | 1.24 |
| 12 | 6.50 | 14.10 | 0.50 | 13.40 | 3.24 | 0.24 | 3.88 | 2.23 |
| 13 | 5.40 | 24.80 | 0.50 | 15.00 | 2.28 | 0.16 | 3.31 | 2.09 |
| 14 | 6.50 | 7.80 | 0.40 | 23.30 | 1.92 | 0.08 | 3.86 | 1.71 |
| 15 | 6.10 | 19.60 | 2.10 | 25.50 | 1.83 | 0.46 | 2.88 | 3.39 |
| 16 | 9.70 | 8.70 | 0.90 | 17.80 | 2.52 | 0.16 | 3.29 | 1.20 |
| 17 | 6.70 | 11.00 | 0.20 | 21.80 | 1.10 | 0.06 | 4.00 | 1.96 |
| 18 | 6.10 | 13.10 | 1.00 | 25.20 | 2.27 | 0.23 | 3.42 | 2.57 |
| 19 | 6.40 | 16.20 | 0.40 | 0.00 | 2.00 | 0.06 | 4.81 | 1.83 |
| 20 | 15.50 | 12.40 | 0.80 | 17.00 | 2.97 | 0.20 | 3.46 | 1.57 |
| 21 | 5.80 | 16.90 | 0.20 | 4.70 | 1.61 | 0.12 | 1.80 | 0.36 |
| 22 | 5.20 | 20.80 | 0.40 | 14.10 | 1.55 | 0.07 | 2.41 | 1.07 |
| 23 | 6.40 | 14.00 | 0.80 | 12.40 | 3.53 | 0.28 | 4.03 | 1.55 |
| 24 | 8.30 | 24.20 | 1.30 | 21.00 | 2.40 | 0.14 | 4.29 | 2.87 |
| 25 | 8.20 | 36.30 | 2.30 | 16.20 | 2.58 | 0.40 | 3.66 | 1.81 |
| 26 | 4.80 | 48.00 | 1.20 | 34.50 | 2.07 | 0.10 | 4.33 | 3.42 |
| 27 | 9.00 | 43.20 | 1.80 | 23.20 | 2.16 | 0.48 | 3.46 | 3.56 |
| 28 | 8.20 | 28.50 | 2.60 | 30.30 | 3.03 | 0.46 | 3.96 | 3.63 |
| 29 | 4.80 | 24.90 | 0.60 | 18.40 | 1.85 | 0.09 | 2.24 | 0.86 |
