# Supplementary material for: Correlation of the total superoxide dismutase activity between joint fluid and synovium in end-stage knee osteoarthritis
Source: Sci Rep. 2024 May 27;14:12093. doi: 10.1038/s41598-024-62614-x (PMC11130189; doi:10.1038/s41598-024-62614-x)
Supplement: Supplementary file 5 — Supplementary Legends. [file 41598_2024_62614_MOESM5_ESM.docx]

**Correlation of the total superoxide dismutase activity between joint fluid and synovium in end-stage knee osteoarthritis**

Masato Koike, Hidetoshi Nojiri, Hiroaki Kanazawa, Mamiko Sawa, Kei Miyagawa, Hiroto Yamaguchi, Yoshiyuki Iwase, Hisashi Kurosawa, Kazuo Kaneko, Muneaki Ishijima

**Supplementary Figures**

**Supplementary Figure S1.** Correlation between SOD1 levels and total SOD activity and between SOD2 and total SOD activity. (a) Correlation between SOD1 levels and total SOD activity in the cartilage (n = 29, Pearson’s correlation). (b) Correlation between SOD1 levels and total SOD activity in the synovium (n = 29, Pearson’s correlation). (c) Correlation between SOD2 levels and SOD2 activity in the cartilage (n = 29, Spearman’s correlation). (d) Correlation between SOD2 levels and SOD2 activity in the synovium (n = 29, Pearson’s correlation). The human SOD1 ELISA Kit (RayBiotech, Inc., USA) was used to measure SOD1 levels. The human SOD2 ELISA Kit (Abnova, USA) was used to measure SOD2 levels. (e) Comparison of total SOD activity in the cartilage and synovium (n = 29, paired t-test, ***P* < 0.01). Error bars represent mean ± standard deviation. (f) Comparison of SOD2 activity in the cartilage and synovium (n = 29, paired t-test, ***P* < 0.01). Error bars represent mean ± standard deviation.

**Supplementary Figure S2.** (a) SOD activity in the cartilage and synovium. (b) SOD protein content in the cartilage and synovium. Center lines show the medians. Box limits indicate the 25th and 75th percentiles, as determined using R software. Whiskers extend to 1.5 times the interquartile range from the 25th and 75th percentiles. Outliers are represented by dots. Data points are plotted as open circles. n = 29 sample points. See Supplementary Table S3 for box plot statistics and Supplementary Table S4 for individual data.

**Supplementary Figure S3.** Differences in joint fluid total SOD activity with and without centrifugation. Total SOD activity was evaluated in the joint fluid of patients with end-stage knee osteoarthritis (OA). There was no difference in total SOD activity due to different centrifugation treatments.
